# Peak Edema Extension Distance: An Edema Measure Independent from Hematoma Volume Associated with Functional Outcome in Intracerebral Hemorrhage

**DOI:** 10.1007/s12028-023-01886-z

**Published:** 2023-11-29

**Authors:** Antje Giede-Jeppe, Stefan T. Gerner, Jochen A. Sembill, Joji B. Kuramatsu, Stefan Lang, Hannes Luecking, Dimitre Staykov, Hagen B. Huttner, Bastian Volbers

**Affiliations:** 1 Department of Neurology, University of Erlangen-Nuremberg, Schwabachanlage 6, 91054 Erlangen, Germany; 2 Department of Neurology, University of Gießen, Gießen, Germany; 3 Department of Neuroradiology, University of Erlangen-Nuremberg, Erlangen, Germany; 4 Neurological Hospital Eisenstadt, Eisenstadt, Austria

**Keywords:** EED, ICH, Edema, PHE, Early EED increase

## Abstract

**Background:**

Our objective was to test the association between hematoma volume and long-term (> 72 h) edema extension distance (EED) evolution and the association between peak EED and early EED increase with functional outcome at 3 months in patients with intracerebral hemorrhage (ICH).

**Methods:**

This retrospective cohort study included patients with spontaneous supratentorial ICH between January 2006 and January 2014. EED, an edema measure defined as the distance between the hematoma border and the outer edema border, was calculated by using absolute hematoma and edema volumes. We used multivariable logistic regression accounting for age, ICH volume, and location and receiver operating characteristic analysis for assessing measures associated with functional outcome and EED evolution. Functional outcome after 3 months was assessed by using the modified Rankin Scale (0–3 = favorable, 4–6 = unfavorable). To identify properties associated with peak EED multivariable linear and logistic regression analyses were conducted.

**Results:**

A total of 292 patients were included. Median age was 70 years (interquartile range [IQR] 62–78), median ICH volume on admission 17.7 mL (IQR 7.9–40.2), median peak perihemorrhagic edema (PHE) volume was 37.5 mL (IQR 19.1–60.6), median peak EED was 0.67 cm (IQR 0.51–0.84) with an early EED increase up to 72 h (EED_72–0_) of 0.06 cm (− 0.02 to 0.15). Peak EED was found to be independent of ICH volume (*R*^2^ = 0.001, *p* = 0.6). In multivariable analyses, peak EED (odds ratio 0.224, 95% confidence interval [CI] [0.071–0.705]) and peak PHE volume (odds ratio 0.984 [95% CI 0.973–0.994]) were inversely associated with favorable functional outcome at 3 months. Receiver operating characteristic analysis identified a peak PHE volume of 26.8 mL (area under the curve 0.695 [95% CI 0.632–0.759]; *p* ≤ 0.001) and a peak EED of 0.58 cm (area under the curve 0.608 [95% CI 0.540–0.676]; *p* = 0.002) as best predictive values for outcome discrimination.

**Conclusions:**

Compared with absolute peak PHE volume, peak EED represents a promising edema measure in patients with ICH that is largely hematoma volume-independent and nevertheless associated with functional outcome.

**Supplementary Information:**

The online version contains supplementary material available at 10.1007/s12028-023-01886-z.

## Introduction

Edema evolution, particularly perihemorrhagic edema (PHE), following intracerebral hemorrhage (ICH) is complex and still incompletely understood [1]. Accumulating evidence suggests that inflammatory mechanisms contribute to ICH-induced secondary brain injury, which is mainly characterized by an immediate release of inflammatory mediators after ICH onset [1, 2]. This inflammatory activation conducted by leukocytes, macrophages, microglia, and astrocytes is sustained for several days [3]. Pivotal pathophysiological roles [4, 5] refer to activated macrophages and microglia contributing to blood–brain barrier injury, edema evolution, and cell death in ICH [6, 7].

In ICH, PHE develops rapidly and increases over several days reaching its maximum within 2 weeks [8, 9]. PHE evolution is linked to unfavorable functional outcome [10–13]. PHE serves as a key radiological surrogate for secondary injury and inflammation following ICH [2, 14, 15] using noncontrast computed tomography (CT) [16, 17]. Because absolute ICH and PHE volumes are closely correlated [11, 16, 18], changes in hematoma volume involve subsequent changes in PHE volume. In clinical ICH, hematoma volume itself is highly variable, therefore absolute PHE volume respectively. This severely impairs the analysis of properties other than hematoma volume associated with edema evolution and thus may require large sample sizes to demonstrate possible treatment effects on edema evolution in clinical trials. To address this issue, a novel PHE measure, the edema extension distance (EED), has been described [19–21]. So far, no association with hematoma volume could be found in acute settings up to 72 h after onset, and data beyond 72 h are not available [22]. Understanding the baseline determinants of EED, e.g., underlying inflammatory mechanisms and its association with clinical outcomes beyond 72 h, is required to establish the utility of EED in ICH clinical trials [21]. In this study, we assessed pathophysiological and outcome-related properties of long-term (> 72 h) EED evolution.

## Methods

### Patients and Inclusion Criteria

The study retrospectively included all patients diagnosed with spontaneous supratentorial ICH who were admitted between January 2006 and January 2014 from a prospectively organized institutional database. ICH was defined as spontaneous if exclusively related to hypertension or amyloid angiopathy. Patients with secondary ICH were excluded. Inclusion criteria were at least two consecutive CT scans over a period of at least 72 h, no withdrawal of care within 24 h after admission, an available modified Rankin Scale (mRS) score after 3 months, and a baseline mRS < 4. Patients with surgical hematoma evacuation were excluded. Absolute PHE volume data derived from this data set have been previously published [23].

### Standard Protocol Approvals, Registrations, and Patient Consents

Ethical approval was obtained by the institutional ethics committee.

### Data Collection

We obtained age [14], mRS prior to symptom onset, National Institutes of Health Stroke Scale score (NIHSS) on admission [14], and other parameters associated with functional outcome after ICH such as fever burden (defined as number of days with peak temperature ≥ 38 °C) up to day 12 [24] and serum inflammatory measures [25] including lymphocytes, neutrophils on admission, and within hospital stay.

Standardized mailed questionnaires were used for the assessment of comorbidities and functional status prior to symptom onset if these data were not obtained during hospital stay. Time between symptom onset and admission was assessed as an important predictor of hematoma expansion [26]. Day 1 was defined as the day of admission.

### Neuroimaging and Volumetric Assessment of EED and ICH

Computed tomography scans were conducted with a fourth-generation CT scanner (Somatom 64 or Somatom AS + 128; Siemens Healthcare, Erlangen). Each scan consisted of either a multislice spiral CT data set, 10–12 slices of 4.8 mm thickness for the skull base and 10–12 slices of 7.2 mm thickness for the cerebrum (Somatom 64), or 22–25 slices of 4.8 mm thickness for the entire brain (Somatom AS+) using the orbitomeatal plane. As described elsewhere [16], a validated semiautomatic volumetric algorithm was used for assessment of ICH and PHE volume. In brief, a region of interest was manually drawn to define the location of the hematoma and the surrounding edema. Within this region of interest, the algorithm (Leonardo V; Siemens Healthcare) calculated the respective volume based on the following Hounsfield-Unit (HU) thresholds: 5–33 HU (edema) and 44–100 HU (hematoma).

As described by Parry-Jones, the EED represents the difference between the radius r€ of a sphere equal to the combined volume of PHE plus ICH and the radius r(h) of a sphere equal to the volume of the ICH alone (average ICH-rounding “thickness” of edema in cm). *EED –€(e)—r(h); *$r(e)= \sqrt[3]{\frac{PHE vol+ ICH vol}{\frac{4}{3} \pi}}$*; *$r(h)= \sqrt[3]{\frac{ICH vol}{\frac{4}{3} \pi}}$; EED = $\sqrt[3]{\frac{PHE vol+ICH vol}{\frac{4}{3} \pi} }-\sqrt[3]{\frac{ICH vol}{\frac{4}{3} \pi}}$ [22]. Early temporal EED changes up to 72 h after admission were calculated from the PHE and ICH volumes at baseline (EED_0_) and maximum EED up to day 3 EED_72–0_ (= − ED_72_–EED_0_) as published before [22]. Peak EED was defined as the maximum distance, measured in centimeters, in any of the available CT scans. CT scans at different time points were merged to time slots (days 1, 2–3, 4–6, 7–9, and 10–12) as available for better comparison. Additionally, intraventricular hemorrhage and hematoma volume on admission [27] as well as peak PHE volume and secondary hematoma enlargement (hematoma expansion) volume were recorded. Peak PHE volume was defined as the maximum volume, measured in milliliters, in any of the available CT scans. To account for accuracy of measurements [28, 29], hematoma expansion was defined as a hematoma volume increase ≥ 5 mL between two CT scans [30].

### Outcome

Functional outcome was evaluated using the mRS scoore 3 months after ICH onset. Two physicians, trained and certified for data collection, conducted a semiquantitative phone interview or mailed standardized questionnaires for mRS score 3 months after ICH onset [31]. Favorable functional outcome was defined and dichotomized as mRS score of 0–3, and unfavorable functional outcome as mRS score of 4–6 [32]. In cases of insufficient data retrieval, we contacted primary care physicians.

### Statistics

Statistical analyses were performed using the IBM SPSS Statistics 24 software package (IBM Corporation, Armonk, NY). The significance level was set at *α* = 0.05 and statistical tests were two-sided. Missing data regarding baseline characteristics, neuroimaging, or functional outcome led to the exclusion of patients. Categorical variables were presented as frequency and percentage, and Pearson *χ*^2^ test and Fisher’s exact test were used to compare between these groups. For continuous variables, the Kolmogorov–Smirnov test was used to determine the distribution of data. If data demonstrated normal distribution, data were presented as means ± standard deviations, and Student’s *t*-test was used for comparison. For data without normal distribution, medians and interquartile ranges (IQRs) are shown and the Mann–Whitney *U*-test was used for comparison, as appropriate.

Multiple imputations were used to account for missing laboratory assessments. Illustrated by scatterplots, linear regression was conducted to reveal possible correlations between peak EED, early EED increase up to day 3, peak PHE volume, and hematoma volume. To identify measures independently associated with unfavorable outcome at 3 months, peak EED distance and early EED increase up to day 3, we calculated linear regression and stepwise forward inclusion multivariable logistic regression models including relevant measures showing a statistical trend (*p* < 0.1) in prior univariate testing. A receiver operating characteristic (ROC) curve and Youden’s J statistic were used to identify best cutoff values for peak PHE, peak EED, and early EED increase [33] to discriminate between favorable and unfavorable functional outcome. To identify factors other than hematoma volume associated with EED evolution we performed univariate and multivariable linear regression analyses. Sensitivity analyses included only patients with available imaging up to (1) day 7–9 (or longer) and (2) day 10–12 (or longer) to assess possible bias due to the nonstandardized imaging time points in our retrospective cohort. For a further sensitivity analysis, we included EED at day 7–9 and day 10–12 into our multivariable logistic regression model instead of peak EED. We additionally performed c-statistics to assess the predictive value of EED at specific time points (day 7–9 and day 10–12) in relation to functional outcome compared with the peak EED, which may occur at different time points depending on patients’ characteristics.

## Results

### Patient Characteristics

As published before [23] 292 patients were included for analysis. Median age was 70 years (IQR 62–78). A total of 131 were women (45%). Median ICH volume on admission was 17.7 mL (IQR 7.9–40.2), with an additional intraventricular hemorrhage in 159/292 patients (54%). ICH was associated with severe neurologic deficits on admission (NIHSS 13 [IQR6–21]). We identified an early EED increase of 0.06 cm (− 0.02 to 0.15) cm up to day 3 (EED_72–0_) with a peak EED of 0.67 (IQR 0.51–0.84) cm. Peak PHE volume was 37.5 mL (IQR 19.1–60.6), as published before [23]. In the overall cohort, peak EED was found at a median of day 7 after admission (IQR 3–12), whereas it was noted earlier in patients with favorable outcome (median day 6 (IQR 2–10) compared with patients with unfavorable outcome (median day 9 [IQR 4–13], *p* < 0.001). Thirty-four patients (12%) had imaging data up to 72 h. For 258 patients (88%), imaging data beyond 72 h was available (up to day 7–9, *n* = 49; up to day 10–12, *n* = 61; up to day 13–15, *n* = 38; beyond day 15, *n* = 70). Patients with unfavorable outcome received neuroimaging up to median day 10–12 (IQR day 7–9 to day 16–19), whereas patients with favorable outcome received imaging up to median day 7–9 (IQR day 4–6 to day 10–12), *p* < 0.001.

### Relationship Between Peak EED, Peak PHE Volume, and Hematoma Volume

**Fig. 1:**
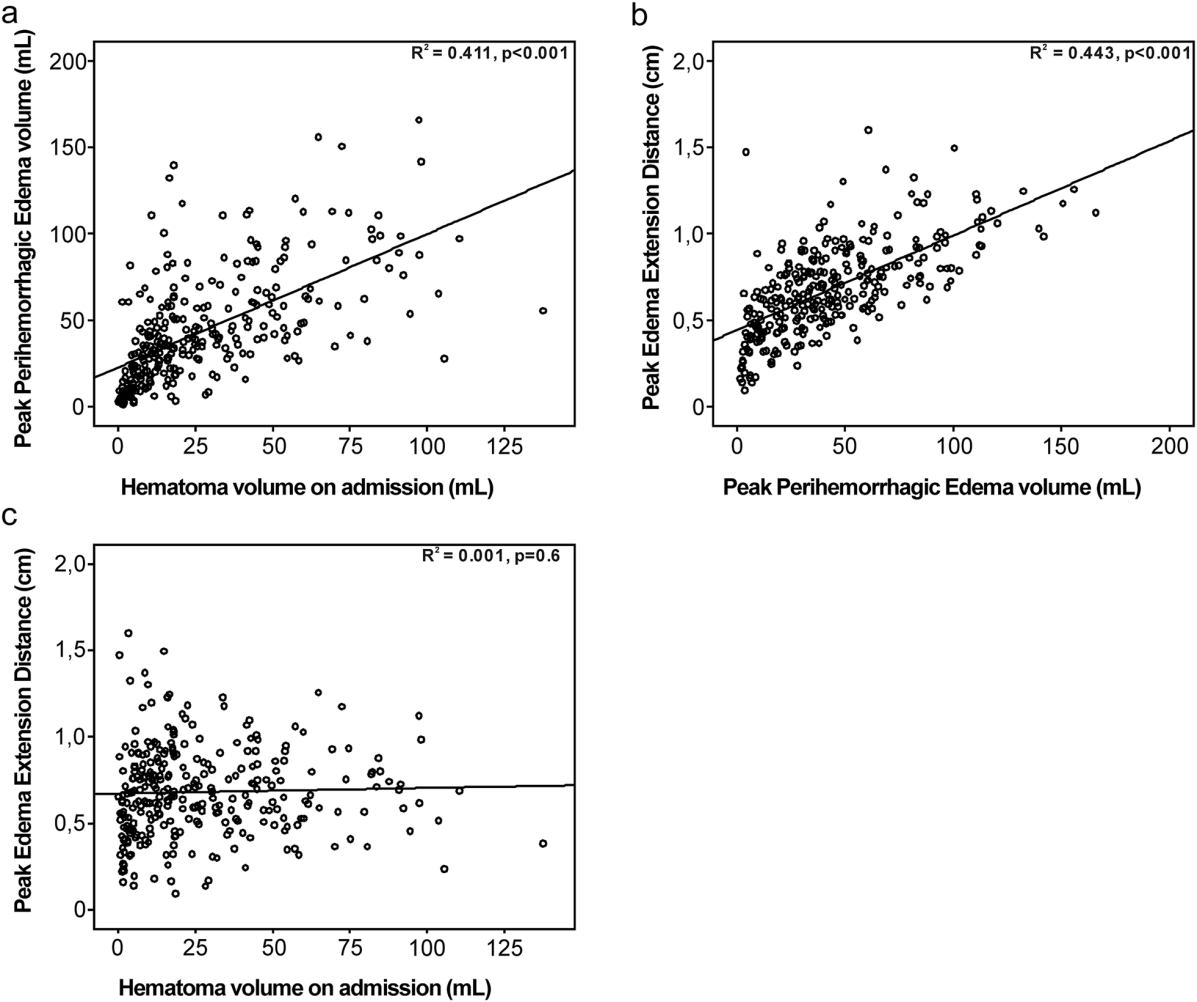
Scatterplot: correlation of peak EED, peak PHE volume, and hematoma volume. Figure 1 demonstrates a positive correlation between peak PHE and ICH hematoma volume on admission (R^2^ = 0.411; *p* < 0.001) (**a**) and peak EED and peak PHE (R^2^ = 0.443, *p* < 0.001). Peak EED showed no correlation with ICH volume (**b**) (**c**, R^2^ = 0.001, *p* = 0.6). EED, edema extension distance, ICH, intracerebral hemorrhage, PHE, perihemorrhagic edema

The relationship between peak EED, peak PHE volume and hematoma volume is demonstrated by scatterplot. Figure 1a–c demonstrates a positive correlation between (Fig. 1a) absolute peak PHE volume and ICH hematoma volume on admission (*R*^2^ = 0.411; *p* < 0.001) and (Fig. 1b) peak EED and absolute peak PHE volume (*R*^2^ = 0.443, *p* < 0.001). There was no association of peak EED with ICH volume (Fig. 1c; *R*^2^ = 0.001, *p* = 0.6).

### EED is Associated with Functional Outcome in Patients with ICH

As previously published [23], a total of 185/292 patients with ICH (63%) showed an unfavorable functional outcome after 3 months. Peak EED was significantly increased in patients with unfavorable functional outcome (0.70 cm [0.54–0.89]; *p* = 0.002) versus in patients with favorable outcome (0.59 cm [0.44–0.78]; *p* = 0.002). Early EED increase up to day 3 (EED_72–0_) was associated with an unfavorable (mRS 4–6) discharge status (0.072 cm [− 0.012 to 0.166], *n* = 203 vs. 0.030 cm [− 0.028 to 0.117] with favorable [mRS 0–3] discharge status, *n* = 89; *p* = 0.043). Detailed characteristics are depicted in Table 1 and Supplemental Table 1. Adjusted multivariable logistic analyses revealed peak EED (odds ratio [OR]: 0.224 95% CI [0.071–0.705] *p* = 0.011, Table 2) and peak PHE volume (OR: 0.984 [95% CI 0.973–0.994] *p* = 0.002; adjusted for identical variables) [23] to be inversely associated with favorable functional outcome at 3 months. Parameters as clinical state on admission measured by NIHSS, age, hematoma volume on admission, and intraventricular hemorrhage were also independently associated with functional outcome (Table 2).

**Table 1:** Characteristics in patients with ICH with favorable and unfavorable functional outcome on day 90

Spontaneous ICH (*n* = 292)	Favorable day 90 outcome (mRS 0–3, *n* = 107)	Unfavorable day 90 outcome (mRS 4–6, *n* = 185)	*p* Value
Peak edema extension distance, median (IQR) (cm)	0.59 (0.44–0.78)	0.70 (0.54–0.89)	0.002*
Early edema extension distance increase up to day 3, median (IQR) (cm)	0.05 (− 0.02 to 0.13)	0.07 (− 0.02 to 0.16)	0.330
Lymphocytes admission, 10^6^/μL	1.3 (1.0–1.8)	1.4 (1.0–2.1)	0.446
Lymphocytes D2/3, 10^6^/μL	1.3 (0.9–1.7)	1.0 (0.8–1.4)	0.007*
Neutrophiles admission, 10^6^/μL	6.3 (4.9–8.4)	6.6 (4.7–8.5)	0.965
Neutrophiles D2/3, 10^6^/μL	6.8 (4.6–8.9)	7.7 (5.7–9.9)	0.012*
Neutrophil-lymphocyte ratio admission	5.1 (2.9–8.1)	5.0 (3.0–8.7)	0.686
Neutrophil-lymphocyte ratio D2/3	4.3 (2.9–7.6)	4.9 (3.7–7.7)	0.029*
Fever burden admission (t > 38 °C)	0 (0)	0 (0)	0.453
Fever burden D2 (t ≥ 38 °C)	0 (0–1)	0 (0–1)	0.674
Fever burden D3 (t ≥ 38 °C)	0 (0–1)	0 (0–1)	0.018*

D2/3, on days 2–3, D2, day 2, D3, day 3 (admission = day 1), ICH, intracerebral hemorrhage, IQR, interquartile range, NIHSS, Nation Institutes of Health Stroke Scale, mRS, modified Rankin Scale (range 0, no deficit, to 6, death), t, temperature

*Indicates statistical significance

**Table 2:** Multivariable logistic regression analysis of parameters associated with favorable functional outcome

Favorable functional outcome (mRS = 0–3)	Odds ratio	95% CI	*p* value
Age (y)	0.942	(0.918–0.967)	< 0.0001*
Hematoma volume on admission (mL)	0.982	(0.969–0.996)	0.010*
Intraventricular hemorrhage	0.379	(0.205–0.700)	0.002*
NIHSS on admission	0.923	(0.894–0.953)	< 0.0001*
Peak edema extension distance (cm)	0.224	(0.071–0.705)	0.011*

Multivariable logistic regression analysis was calculated for the association with favorable functional outcome defined as a score between 0 and 3 on the modified Rankin Scale. Only parameters showing a statistical trend (*p* < 0.1) in prior univariate testing were included in the multivariable model as recently published [23]. For each parameter, odds ratio, and 95% CIs are provided

CI, Confidence interval, mRS, modified Rankin Scale (range 0, no deficit, to 6, death), NIHSS, National Institues of Health Stroke Scale

*Indicates statistical significance

Sensitivity analysis including only patients with available imaging data up to day 7–9 or longer (*n* = 218) and up to day 10–12 or longer (*n* = 169) revealed comparable results. Peak EED was independently associated with functional outcome after adjustment for age, hematoma volume on admission, intraventricular hemorrhage, and NIHSS on admission (day 7–9: adjusted OR [peak EED, cm] 0.142 [95% CI 0.039–0.515], day 10–12: adjusted OR [peak EED, cm] 0.243 [95% CI 0.059–1.000]).

Including EED at day 7–9 (*n* = 218) instead of peak EED into the multivariable logistic regression model showed an independent association of EED at day 7–9 with day 90 functional outcome (adjusted OR 0.142 [95% CI 0.032–0.486]). EED at day 10–12 (*n* = 169) also showed an independent association with functional outcome at day 90 (adjusted OR 0.168 [95% CI 0.032–0.486], Supplemental Table 5).

### EED Evolution within Hospital Stay: Early EED Increase up to Day 3

**Fig. 2:**
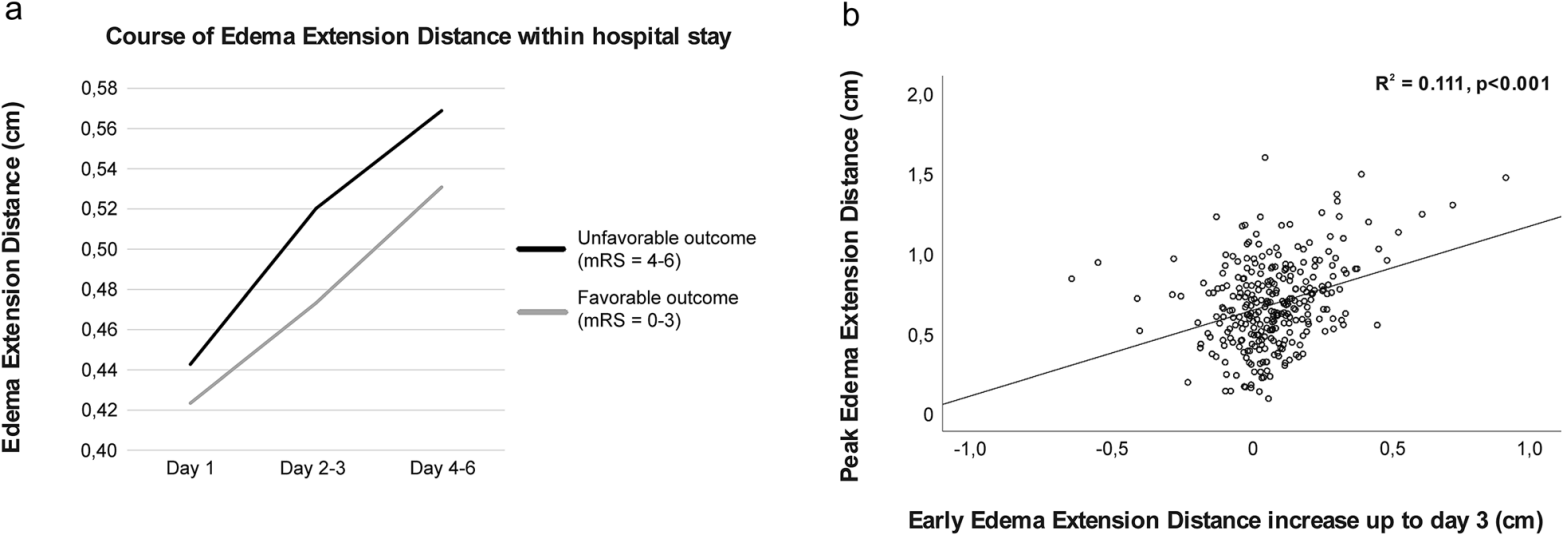
Evolution of early EED increase. **a** EED evolution within hospital stay dependent on day 90 functional outcome, early EED increase (EED_72–0_) up to day 3 (**b**) revealed a positive correlation with peak EED (R^2^ = 0.111, *p* < 0.001). EED, edema extension distance, mRS, modified Rankin Scale

Figure 2a depicts EED evolution in patients with favorable and unfavorable functional outcome at 3 months: Within the first 3 days after ICH onset, patients with unfavorable functional outcome showed a slightly increased EED evolution compared with patients with favorable functional outcome. Within clinical stay beyond day 2/3 the EED increase appears to be less pronounced. Early EED increase up to day 3 (EED_72–0_) revealed a positive correlation with peak EED (Fig. 2a; R^2^ = 0.111, *p* < 0.001).

### Associations of Peak PHE, Early EED Increase up to Day 3, and Peak EED with Unfavorable Functional Outcome at 3 Months

**Fig. 3:**
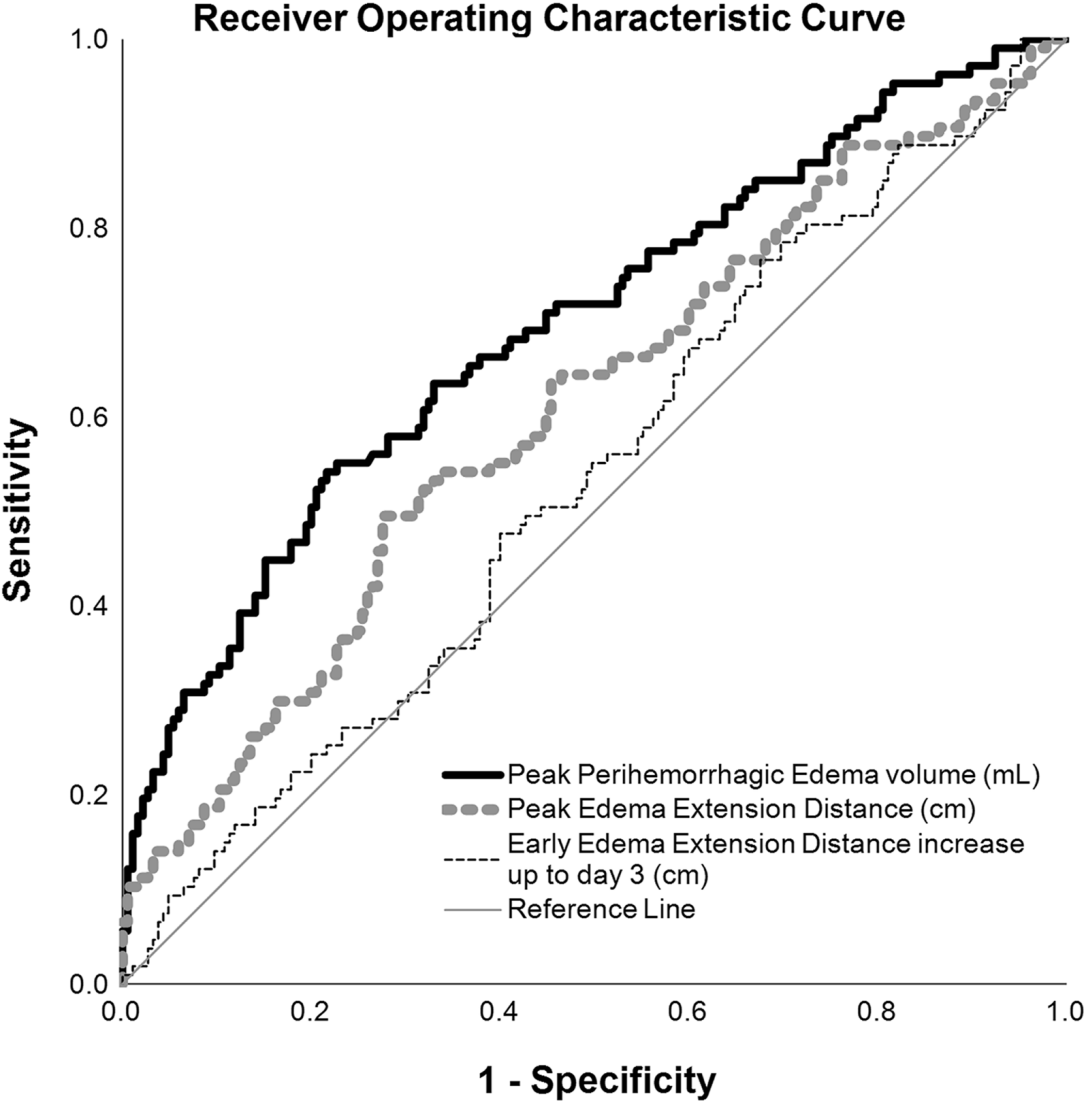
Association of peak perihemorrhagic edema volume (mL), peak edema extension distance (cm), and early edema extension distance increase up to day 3 (cm), with unfavorable functional outcome. ROC curve for prediction of unfavorable functional outcome. ROC plot demonstrated the AUC for unfavorable functional outcome. AUC, area under the curve, ROC, receiver operating characteristic

Among known parameters as peak PHE volume (26.8 mL, area under the curve [AUC] 0.695 [95% CI 0.632–0.759]; *p* < 0.001) [23], ROC analysis identified a peak EED of 0.58 cm as the best cutoff threshold to discriminate between patients with favorable and unfavorable functional outcome after 3 months (AUC = 0.608 [95% CI 0.540–0.676]; *p* = 0.002, Youden’s Index = 0.219; sensitivity, 49.5%; specificity, 72.4%; unfavorable functional outcome: EED_peak_ ≥ 0.58 cm 134/185 [72%] vs. EED_peak_ < 0.58 cm 51/185 [28%]; *p* < 0.001, Fig. 3 and Supplemental Tables 2 and 3).

Early EED increase up to day 3 (EED_72–0_) was not sufficient to discriminate between patients with favorable and unfavorable functional outcome (AUC = 0.534 [95% CI 0.466–0.603]; *p* = 0.330, Youden’s Index = 0.09; sensitivity, 76.6%; specificity, 32.4%; unfavorable outcome: EED_72_ ≥ 0.14 cm 55/185[30%] vs. EED_72_ < 0.14 cm 130/185 [70%]; *p* = 0.125; Fig. 3 and Supplemental Tables 2 and 3). Optimal cutoff values were confirmed for peak PHE before [23]. EED on day 7–9 (AUC 0.568 [95% CI 0.482–0.654], *p* = 0.107) and EED on day 10–12 (AUC 0.544 [95% CI 0.437–0.652], *p* = 0.370) were also insufficient to discriminate between patients with favorable and unfavorable outcome.

### Parameters Associated with Peak EED

Given that peak EED was identified to be independently associated with functional outcome at 3 months, we analyzed parameters associated with EED evolution. In univariate linear testing fever burden (*T* ≥ 38 °C on day 2, *p* = 0.043), and lymphocyte counts on day 2/3 (*p* = 0.004) showed associations with peak EED (Additional file 1: Supplemental Table 3). In multivariable linear analysis fever burden (*T* ≥ 38 °C on day 2, *p* = 0.048) remained independently associated with peak EED. Low lymphocyte counts (*p* = 0.051) and fever burden on day 3 (*p* = 0.074) showed a statistical trend in multivariable testing (Table 3).

**Table 3:** Multivariable linear regression analysis of parameters associated with peak EED

Peak edema extension distance (cm)	Regression coefficient	95% CI	*p* Value
Age (y)	− 0.001	− 0.005 to 0.002	0.409
Prior antiplatelets	− 0.062	− 0.143 to 0.020	0.139
Absolute rebleed volume (mL)	0.002	− 0.001 to 0.005	0.128
Fever burden D2 (t ≥ 38 °C)	0.040	0.000 to 0.079	0.048*
Fever burden D3 (t > 38 °C)	0.077	− 0.008 to 0.162	0.074
Lymphocytes D2/3, 10^6^/µL	0.068	0.000 to 0.136	0.051

Multivariable linear regression analysis was calculated for the association with peak EED. Only parameters showing a statistical trend (*p* < 0.1) in prior univariate linear testing were included in the linear model. For each parameter, risk ratio, and 95% CIs are provided

CI, Confidence interval, D2/3, on days 2–3, D2, on day 2 (admission = day 1), EED, edema extension distance, t, temperature

*Indicates statistical significance

## Discussion

This analysis has shown for the first time that peak EED is a hematoma-volume independent edema measure that is associated with functional outcome measured by mRS at 3 months. We provide further evidence that peak edema depicted as peak EED appears to be superior for outcome discrimination than parameters demonstrating an early EED increase. For peak absolute PHE volume similar results related to functional outcome were published recently [23], denoting that peak absolute PHE volume yields stronger associations with functional outcome than peak EED. However, because absolute PHE volume is strongly associated with ICH volume, EED may represent an interesting and promising hematoma-independent edema measure in future ICH research, which is outcome-related and yet independent from hematoma volume.

Because of feasibility reasons, existing edema research predominantly focuses on early edema evolution up to 48–72 h after symptom onset. In this context, an independent association of early edema increase and functional outcome could be shown [34–36]. The present study did not reveal associations of an early EED increase up to 72 h (EED_72–0_) and functional outcome in univariate testing. Reasons may be the cohort size of the present study and a therefore diminished statistical power as we identified significant associations between early EED increase and unfavorable clinical status at discharge (*p* = 0.043, in univariate testing). However, a weak association with day 90 outcome was existent in the present cohort for absolute early PHE volume increase as published before [23]. Thus, edema parameters reflecting an early edema increase revealed a weaker edema measure in this cohort concerning their association with functional outcome. In line with the previously mentioned effects of early absolute PHE volume increase, this association with functional outcome could also be demonstrated in larger cohorts for early EED increase [22]. Still, our results revealed peak EED as an independent outcome predictor in patients with ICH. Adding to that, ROC analysis relates absolute peak PHE volume closer to functional outcome than peak EED. Thus, it seems that absolute edema volumes and peak edema measures are closer related to outcome than extension distance measures and early edema increase up to 72 h, respectively. Since early edema increase is associated with peak edema independently from the used edema measure (absolute volume [23]/EED [Fig. 2b]), further studies need to elucidate whether the association of early edema evolution vs. peak edema with functional outcome may interact with the cohort size. In addition, our data showed a stronger association between individual patient peak edema (EED) and functional outcome than edema measures at specific time points during hospitalization up to day 12. Although this result may be susceptible to bias because of the non-standardized imaging time points in our study, sensitivity analyses including only patients with continuous imaging data up to day 12 or longer yielded consistent results. In 50% of the patients, peak EED was noted between day 3 and 12. Future studies will need to address this issue in more detail, as it may affect study design in terms of scheduling follow-up imaging time points.

In line with published data [21, 22] limited to early edema evolution < 72 h, we could confirm that peak EED is largely independent of hematoma volume. This may represent an important property of EED compared to absolute edema volume, which is strongly associated with hematoma volume [9]. Compared with absolute PHE volume, Parry-Jones et al. could already confirm that smaller cohorts may be sufficient to show both therapeutic and pathophysiological edema-dependent associations using EED as edema measure because the hematoma dependent interaction is mostly negligible [22]. Thus, this particular approach to employ peak EED as a preferred PHE measure may permit an earlier selection of promising treatments in smaller cohorts to thereby push new findings from bench to bedside.

Inflammatory processes start within hours after ICH onset and sustain over several days with edema as an important pathophysiological component of this inflammatory response to ICH [14]. Natural history studies report peak edema volumes between week 2 and 3 [9], which emphasizes the dynamic character of edema evolution and the need for imaging data beyond 72 h as provided by this data set. Surrogate markers to assess the efficacy of potential anti-inflammatory treatment approaches remain to be established, while edema seems to be a promising candidate [36]. In our cohort, we found associations of especially early systemic inflammatory measures up to day 3 after onset with peak EED. Accordingly, one may speculate that the early inflammatory response may also contribute to edema evolution at later time points. Thus, peak EED (as a hematoma-independent and yet outcome-associated edema measure) may represent such a valuable marker to precisely assess edema-related associations with inflammatory processes as well as the efficacy of anti-inflammatory treatments. Promising anti-inflammatory candidates are, e.g., siponimod [37] or fingolimod [38]. However, efficacy of those treatments remains to be elucidated in randomized controlled trials.

Strengths of the present study include a validated volumetric PHE assessment, the assessment of PHE evolution beyond 72 h up to 10–12 days after symptom onset and a consistent cohort with strict inclusion criteria from a prospectively organized institutional database.

We acknowledge several limitations of this study. It lacks a prospective and multicenter design. Therefore, imaging data were not obtained by standardized scheduling. To address this issue, imaging data were merged according to prespecified time slots, which may impose bias by underestimation of peak EED and to a distorted representation of early EED increase up to day 3 and the time axis of EED evolution. Because only patients with supratentorial ICH were included, these findings cannot be transferred to the entity of infratentorial ICH. Further, patients with early withdrawal of care, patients with less than two CT scans (*n* = 294) and 38 patients with missing data were excluded from the data set, which may impose bias to the reported data. As length of hospital stay and number of CT scans showed an interindividual variation in some patients the real peak EED might have been missed. However, sensitivity analyses including only patients with available continuous imaging data up to day 12 yielded consistent results. In addition, a large proportion of patients had imaging data available up to day 12 or longer. Validation of this methodology was performed up to day 5 after admission. We have to be aware that due to ongoing hematoma degradation a certain inaccuracy may affect EED assessment between day 6 and 12, even though this method adjusts for hematoma degradation. Time between symptom onset and admission did not differ between patients with and without hematoma expansion. However, as this measure differed between individuals, it might impose possible bias in this complex relation among hematoma evolution, EED evolution, outcome, and time. The gold standard to depict PHE on neuroimaging is MRI, which is not feasible for serial follow-up imaging in patients with ICH. Thus, we used a recommended [22] and validated semiautomatic threshold based algorithm [16] to quantify PHE on CT scans.

## Conclusions

Peak EED represents a promising hematoma-independent edema measure in patients with ICH that is independently associated with functional outcome. Compared with absolute peak PHE volume, using peak EED as an edema-related measure may be more suitable to assess edema-dependent processes and therapeutic issues, especially in smaller ICH cohorts.

### Supplementary Information

Below is the link to the electronic supplementary material.

Supplemental Table 1 Abbreviations: ICH = intracerebral hemorrhage, IQR = Interquartile Range, mL = milliliter, mRS = modified Rankin Scale, NIHSS = National Institutes of Health Stroke Scale, VKA = Vitamin-K-Antagonist, y = year. Supplemental Tables 2 and 3: Association of peak PHE volume, peak EED and early EED increase up to day 3 with unfavorable functional outcome. ROC curve for prediction of unfavorable functional outcome. ROC plot demonstrated the AUC for unfavorable functional outcome. The best cutoff value to discriminate between favorable and unfavorable outcome at 3 months was identified at a peak PHE volume of 26.81 mL as recently published23 and a peak EED of 0.58 cm. Obtained cutoff values for early EED increase up to day 3, cm of 0.14 were not sufficient to discriminate between favorable and unfavorable functional outcome. Abbreviations: AUC = area under the curve, cm = centimeter, 95% CI = Confidence Interval, EED = Edema Extension Distance, mL = milliliter, PHE = Perihemorrhagic Edema, YI = Youden’s Index. Supplemental Table 4: Univariate linear regression analysis of parameters associated with peak EED. Univariate linear regression analysis was calculated for the association with peak EED. For each parameter, regression coefficient and 95% confidence interval are provided. Significant findings expressed in boldface type were included into a multivariable model as depicted in Table 3. Abbreviations: 95% CI = Confidence Interval, D2 = day 2, D3 = day 3, D2/3 = day 2-3, EED = Edema Extension Distance, IQR = Interquartile Range, mL = milliliter, mRS = modified Rankin Scale, NIHSS = National Institutes of Health Stroke Scale, myL = microliter, t = temperature, VKA = Vitamin-K-Antagonist, y = year. Supplemental Table 5: Sensitivity Analysis including only patients with continuous neuroimaging up to (1) day 7-9 and (2) day 10-12 assessing the association of peak edema extension distance with outcome. (a) Sensitivity analysis including exclusively patients with continuous neuroimaging up to day 7-9 (or longer). N = 218 unfavorable outcome: n= 149 (68%), favorable outcome = 69 (32%); (Complete cohort 63% vs. 37%). (b) Sensitivity analysis including exclusively patients with continuous neuroimaging up to day 10-12 (or longer). N = 169 unfavorable outcome: n= 121 (72%), favorable outcome = 48 (28%); (Complete cohort 63% vs. 37%). Sensitivity analysis including edema extension distance on (1) day 7-9 and (2) day 10-12 instead of peak edema extension distance into the multivariable logistic regression model. (a) Sensitivity analysis including EED days 7-9 instead of peak EED into the multivariable logistic regression predicting functional outcome. N = 218; unfavorable outcome: n= 149 (68%), favorable outcome = 69 (32%); (Complete cohort 63% vs. 37%). (b) Sensitivity analysis including EED days 10-12 instead of peak EED into the multivariable logistic regression predicting functional outcome. N = 169; unfavorable outcome: n= 121 (72%), favorable outcome = 48 (28%); (Complete cohort 63% vs. 37%). Abbreviations: 95% CI = Confidence Interval, cm = centimeter, EED = Edema Extension Distance, IQR = Interquartile Range mL = milliliter, mRS = modified Rankin Scale, NIHSS = National Institutes of Health Stroke Scale, VKA = Vitamin-K-Antagonist, y = year (DOCX 16 kb)

## References

[1] Urday S, Kimberly WT, Beslow LA (2015). Targeting secondary injury in intracerebral haemorrhage–perihaematomal oedema. Nat Rev Neurol.

[2] Xi G, Keep RF, Hoff JT (2006). Mechanisms of brain injury after intracerebral haemorrhage. Lancet Neurol.

[3] Wang J (2010). Preclinical and clinical research on inflammation after intracerebral hemorrhage. Prog Neurobiol.

[4] Wan S, Cheng Y, Jin H (2016). Microglia activation and polarization after intracerebral hemorrhage in mice: the role of protease-activated receptor-1. Transl Stroke Res.

[5] Koh YC, Yang G, Lai CS, Weerawatanakorn M, Pan MH (2018). Chemopreventive effects of phytochemicals and medicines on M1/M2 polarized macrophage role in inflammation-related diseases. International Journal of Molecular Sciences.

[6] Bhatia HS, Baron J, Hagl S, Eckert GP, Fiebich BL (2016). Rice bran derivatives alleviate microglia activation: possible involvement of MAPK pathway. J Neuroinflammation.

[7] Taylor RA, Sansing LH (2013). Microglial responses after ischemic stroke and intracerebral hemorrhage. Clin Dev Immunol.

[8] Balami JS, Buchan AM (2012). Complications of intracerebral haemorrhage. Lancet Neurol.

[9] Staykov D, Wagner I, Volbers B (2011). Natural course of perihemorrhagic edema after intracerebral hemorrhage. Stroke.

[10] Zazulia AR, Diringer MN, Derdeyn CP, Powers WJ (1999). Progression of mass effect after intracerebral hemorrhage. Stroke.

[11] Appelboom G, Bruce SS, Hickman ZL (2013). Volume-dependent effect of perihaematomal oedema on outcome for spontaneous intracerebral haemorrhages. J Neurol Neurosurg Psychiatry.

[12] Mayer SA, Sacco RL, Shi T, Mohr JP (1994). Neurologic deterioration in noncomatose patients with supratentorial intracerebral hemorrhage. Neurology.

[13] Murthy SB, Moradiya Y, Dawson J (2015). Perihematomal edema and functional outcomes in intracerebral hemorrhage: influence of hematoma volume and location. Stroke.

[14] Qureshi AI, Mendelow AD, Hanley DF (2009). Intracerebral haemorrhage. Lancet.

[15] Ziai WC (2013). Hematology and inflammatory signaling of intracerebral hemorrhage. Stroke.

[16] Volbers B, Staykov D, Wagner I (2011). Semi-automatic volumetric assessment of perihemorrhagic edema with computed tomography. Eur J Neurol.

[17] Mould WA, Carhuapoma JR, Muschelli J (2013). Minimally invasive surgery plus recombinant tissue-type plasminogen activator for intracerebral hemorrhage evacuation decreases perihematomal edema. Stroke.

[18] Arima H, Wang JG, Huang Y (2009). Significance of perihematomal edema in acute intracerebral hemorrhage: the INTERACT trial. Neurology.

[19] Wu TY, Putaala J, Sharma G (2017). Persistent Hyperglycemia Is Associated With Increased Mortality After Intracerebral Hemorrhage. Journal of the American Heart Association.

[20] Wu TY, Sharma G, Strbian D (2017). Natural history of perihematomal edema and impact on outcome after intracerebral hemorrhage. Stroke.

[21] Hurford R, Vail A, Heal C (2019). Oedema extension distance in intracerebral haemorrhage: association with baseline characteristics and long-term outcome. Eur Stroke J.

[22] Parry-Jones AR, Wang X, Sato S (2015). Edema extension distance: outcome measure for Phase II clinical trials targeting edema after intracerebral hemorrhage. Stroke.

[23] Volbers B, Giede-Jeppe A, Gerner ST (2018). Peak perihemorrhagic edema correlates with functional outcome in intracerebral hemorrhage. Neurology.

[24] Schwarz S, Hafner K, Aschoff A, Schwab S (2000). Incidence and prognostic significance of fever following intracerebral hemorrhage. Neurology.

[25] Giede-Jeppe A, Bobinger T, Gerner ST (2016). Lymphocytopenia Is an independent predictor of unfavorable functional outcome in spontaneous intracerebral hemorrhage. Stroke.

[26] Brouwers HB, Chang Y, Falcone GJ (2014). Predicting hematoma expansion after primary intracerebral hemorrhage. JAMA Neurol.

[27] Broderick JP, Brott TG, Duldner JE, Tomsick T, Huster G (1993). Volume of intracerebral hemorrhage: A powerful and easy-to-use predictor of 30-day mortality. Stroke.

[28] Ziai W, Carhuapoma JR, Nyquist P, Hanley DF (2016). Medical and surgical advances in intracerebral hemorrhage and intraventricular hemorrhage. Semin Neurol.

[29] Webb AJ, Ullman NL, Morgan TC (2015). Accuracy of the ABC/2 score for intracerebral hemorrhage: systematic review and analysis of MISTIE, CLEAR-IVH, and CLEAR III. Stroke.

[30] Hanley DF, Thompson RE, Muschelli J (2016). Safety and efficacy of minimally invasive surgery plus alteplase in intracerebral haemorrhage evacuation (MISTIE): a randomised, controlled, open-label, phase 2 trial. Lancet Neurol.

[31] van Swieten JC, Koudstaal PJ, Visser MC, Schouten HJ, van Gijn J (1988). Interobserver agreement for the assessment of handicap in stroke patients. Stroke.

[32] Rankin J (1957). Cerebral vascular accidents in patients over the age of 60. I. General considerations. Scott Med J.

[33] Hanley JA, McNeil BJ (1982). The meaning and use of the area under a receiver operating characteristic (ROC) curve. Radiology.

[34] Yang J, Arima H, Wu G (2015). Prognostic significance of perihematomal edema in acute intracerebral hemorrhage: pooled analysis from the intensive blood pressure reduction in acute cerebral hemorrhage trial studies. Stroke.

[35] Murthy SB, Urday S, Beslow LA (2016). Rate of perihaematomal oedema expansion is associated with poor clinical outcomes in intracerebral haemorrhage. J Neurol Neurosurg Psychiatry.

[36] Selim M, Norton C (2020). Perihematomal edema: implications for intracerebral hemorrhage research and therapeutic advances. J Neurosci Res.

[37] Bobinger T, Manaenko A, Burkardt P (2019). Siponimod (BAF-312) attenuates perihemorrhagic edema and improves survival in experimental intracerebral hemorrhage. Stroke.

[38] Zhu Z, Fu Y, Tian D (2015). Combination of the immune modulator fingolimod with alteplase in acute ischemic stroke: A pilot trial. Circulation.

