# Supplementary material for: Peak Edema Extension Distance: An Edema Measure Independent from Hematoma Volume Associated with Functional Outcome in Intracerebral Hemorrhage
Source: Neurocrit Care. 2023 Nov 29;40(3):1089–98. doi: 10.1007/s12028-023-01886-z (PMC11147861; doi:10.1007/s12028-023-01886-z)
Supplement: Supplementary file 1 — Supplemental Table 1 Abbreviations: ICH = intracerebral hemorrhage, IQR = Interquartile Range, mL = milliliter, mRS = modified Rankin Scale, NIHSS = National Institutes of Health Stroke Scale, VKA = Vitamin-K-Antagonist, y = year. Supplemental Tables 2 and 3: Association of peak PHE volume, peak EED and early EED increase up to day 3 with unfavorable functional outcome. ROC curve for prediction of unfavorable functional outcome. ROC plot demonstrated the AUC for unfavorable functional outcome. The best cutoff value to discriminate between favorable and unfavorable outcome at 3 months was identified at a peak PHE volume of 26.81 mL as recently published23 and a peak EED of 0.58 cm. Obtained cutoff values for early EED increase up to day 3, cm of 0.14 were not sufficient to discriminate between favorable and unfavorable functional outcome. Abbreviations: AUC = area under the curve, cm = centimeter, 95% CI = Confidence Interval, EED = Edema Extension Distance, mL = milliliter, PHE = Perihemorrhagic Edema, YI = Youden’s Index. Supplemental Table 4: Univariate linear regression analysis of parameters associated with peak EED. Univariate linear regression analysis was calculated for the association with peak EED. For each parameter, regression coefficient and 95% confidence interval are provided. Significant findings expressed in boldface type were included into a multivariable model as depicted in Table 3. Abbreviations: 95% CI = Confidence Interval, D2 = day 2, D3 = day 3, D2/3 = day 2-3, EED = Edema Extension Distance, IQR = Interquartile Range, mL = milliliter, mRS = modified Rankin Scale, NIHSS = National Institutes of Health Stroke Scale, myL = microliter, t = temperature, VKA = Vitamin-K-Antagonist, y = year. Supplemental Table 5: Sensitivity Analysis including only patients with continuous neuroimaging up to (1) day 7-9 and (2) day 10-12 assessing the association of peak edema extension distance with outcome. (a) Sensitivity analysis including exclusively patients [file 12028_2023_1886_MOESM1_ESM.docx]

**Supplemental Material:**

**Supplemental table 1**

| **Spontaneous ICH (n = 292)** | **Favorable day 90 outcome**  **(mRS 0-3, n = 107)** | **Unfavorable day 90 outcome**  **(mRS 4-6, n = 185)** | **p Value** |
| --- | --- | --- | --- |
| ***As previously published by Volbers et al.*** | | | |
| Age, y, median (IQR) | 69 (59–76) | 72 (64–79) | **0.007** |
| Male sex, n (%) | 57 (53) | 104 (56) | 0.714 |
| NIHSS on admission, median (IQR) | 5 (2–11) | 16 (11–35) | **<0.001** |
| Baseline mRS, median (IQR) | 1 (0–2) | 1 (0–2) | **0.003** |
| Intraventricular hemorrhage, n (%) | 38 (36) | 121 (65) | **<0.001** |
| Prior Antiplatelets | 30 (28) | 58 (31) | **0.598** |
| Prior Oral Anticoagulation (VKA) | 16 (15) | 34 (18) | **0.521** |
| Prior Statin medication | 28 (26) | 36 (20) | **0.189** |
| ICH-volume, mL, median (IQR) | 10.6 (4.9–25.6) | 21.4 (11.2–44.4) | **<0.001** |
| Peak perihemorrhagic edema volume, mL, median (IQR) | 23.8 (9.1–45.3) | 42.6 (28.1–67.4) | **<0.001** |

**Supplemental table 2**

| **Receiver Operating Characteristic Curve** | **AUC** | **CI 95%** | **p Value** |
| --- | --- | --- | --- |
| Peak Perihemorrhagic Volume, mL | 0.695 | (0.632-0.759) | **<0.001** |
| Peak Edema Extension distance, cm | 0.608 | (0.540-0.676) | **0.002** |
| Early Edema Extension Distance increase up to  day 3, cm | 0.534 | (0.466-0.603) | 0.330 |

**Supplemental table 3**

|  | **Cut-off**  **threshold** | **Sensitivity** | **Specificity** | **Sensitivity +**  **Specificity** | **YI** |
| --- | --- | --- | --- | --- | --- |
| Peak Perihemorrhagic  Volume, mL: | 26.81 | 0.542 | 0.784 | 1.326 | 0.326 |
| Early Edema Extension  Distance up to day 3, cm: | 0.14 | 0.766 | 0.324 | 1.09 | 0.09 |
| Peak Edema Extension  distance, cm: | 0.58 | 0.495 | 0.724 | 1.219 | 0.219 |

**Supplemental table 4**

| **Peak Edema Extension Distance, cm** | **Regression coefficient** | **95% CI** | **p Value** |
| --- | --- | --- | --- |
| Age, years | -0.002 | -0.005-0 | 0.053 |
| Male sex | 0.014 | -0.048-0.075 | 0.666 |
| Intraventricular Hemorrhage | -0.001 | -0.063-0.060 | 0.969 |
| Location lobar | -0.024 | -0.086-0.038 | 0.448 |
| Hematoma volume on admission, mL | 0 | -0.001-0.001 | 0.673 |
| Absolute rebleed Volume, mL | 0.002 | (0.000-0.005) | 0.084 |
| Peak Hematoma volume, mL | 0.001 | 0-0.002 | 0.274 |
| Hypertension | -0.083 | -0.192-0.025 | 0.133 |
| Prior Antiplatelets | -0.066 | -0.132-0 | 0.050 |
| Prior Oral Anticoagulation (VKA) | -0.007 | -0.089-0.074 | 0.858 |
| Prior Statin medication | -0.031 | -0.105-0.043 | 0.405 |
| Fever burden Admission (t > 38°C) | -0.015 | -0.083-0.053 | 0.659 |
| Fever burden D2 (t > 38°C) | 0.081 | (0.003-0.160) | **0.043** |
| Fever burden D3 (t > 38°C) | 0.049 | -0.009-0.016 | 0.097 |
| Neutrophiles Admission, 10^6/myL | 0.001 | -0.010-0.012 | 0.828 |
| Neutrophiles D2/3, 10^6/myL | 0.001 | -0.005-0.006 | 0.825 |
| Neutrophil-lymphocyte ratio Admission | 0.003 | -0.002-0.008 | 0.313 |
| Neutrophil-lymphocyte ratio D2/3 | -0.001 | -0.007-0.006 | 0.872 |
| Lymphocytes Admission, 10^6/myL | -0.012 | -0.054-0.031 | 0.586 |
| Lymphocytes D2/3, 10^6/myL | 0.084 | (0.028-0.141) | **0.004** |
| Therapeutic Hypothermia | 0.033 | -0.055-0.121 | 0.458 |
